# Soluble transferrin receptor level, inflammation markers, malaria, alpha‐thalassemia and selenium status are the major predictors of hemoglobin in children 6–23 months in Malawi

**DOI:** 10.1002/fsn3.1780

**Published:** 2020-07-16

**Authors:** Samson Gebremedhin

**Affiliations:** ^1^ School of Public Health Addis Ababa University Addis Ababa Ethiopia

**Keywords:** anemia, hemoglobin, inflammation, iron deficiency, malaria, selenium

## Abstract

In sub‐Saharan Africa, nearly three‐fourths of children 6–23 months are anemic. Yet, the underlying causes had not been sufficiently explored. This study, based on data (*n* = 348) extracted from the Malawi Micronutrient Survey–2015/2016 dataset, evaluated the contribution of multiple factors to the hemoglobin status of children 6–23 months. The association between hemoglobin and 19 predictors was assessed using multiple linear regression analysis, and the relative contribution of the covariates was determined based on delta‐*R* ^2^ value. The study found that 43.9% of children were anemic and 76.9% had elevated soluble transferrin receptor (sTfR) levels. Unit changes in serum ferritin (µg/L) and sTfR (mg/L) were associated with 0.01 g/dl rise (*p* = .041) and 0.05 g/dl decline (*p* < .001) in hemoglobin, respectively. Each 1 ng/ml increase in plasma selenium was met with 0.007 g/dl (*p* = .02) rise in hemoglobin. Hemoglobin showed negative relationships with α‐1‐acid glycoprotein (AGP) (*β* = −.339, *p* = .007) and C‐reactive protein (CRP) (*β* = −.014, *p* = .004) and positive association with child's age in months (*β* = .038, *p* = .003) and altitude in meters (*β* = .001, *p* = .015). Children affected by α‐thalassemia (*β* = −.75, *p* < .001), malaria (*β* = −.43, *p* = .029), and fever (*β* = −.39, *p* = .008) had significantly lower hemoglobin levels. On the contrary, nine variables including serum zinc and retinol binding protein were not significant predictors of hemoglobin. sTfR had the highest delta‐*R* ^2^ contribution (9.1%) to hemoglobin variations, followed by inflammation (5.2%), α‐thalassemia (2.5%), age (2.1%), fever (1.9%), and malaria (1.5%). The analysis suggested iron status, inflammation, and malaria were the major predictors of hemoglobin among Malawian infants and young children.

## Graphical abstract

Iron status indicators followed by inflammation markers, alpha‐thalassemia, malaria and selenium status are the major predictors of hemoglobin in children 6–23 months in Malawi.

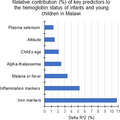

## 1 BACKGROUND

Anemia is a complex disorder characterized by suboptimal hemoglobin or red blood cell mass that results in diminished cellular perfusion (Kraemer & Zimmermann, 2007). Though it is commonly asserted that at least half of the global burden of anemia is attributable to iron deficiency, other micronutrient deficiencies including folate, vitamin B12, vitamin A, and zinc and genetic disorders like sickle cell disease and thalassemia, chronic and infectious diseases, hemolysis or blood loss, bone marrow disorders lead to anemia. Furthermore, the epidemiological significance of the specific causes is likely to vary across populations (de Benoist, McLean, Egli, & Cogswell, 2008; Kraemer & Zimmermann, 2007).

Globally, more than 1.6 billion people, equivalent to a quarter of the world population, are affected by anemia (de Benoist et al., 2008); and preschool children (43%) and pregnant women (38%) take the highest burden (WHO, 2015). While anemia has global public health significance, sub‐Saharan Africa (SSA) and South‐East Asia are disproportionally affected, and in these regions, nearly half of women and two‐thirds of children are anemic (de Benoist et al., 2008). Developing countries also contribute as high as 90% for the global anemia‐related disability (Kassebaum, 2016). Though the prevalence of anemia significantly dropped in the last three decades, the decline had been steady and the Global Nutrition Target for reducing anemia by 50% between 1990 and 2025 is unlikely to be achieved (Kassebaum et al., 2014; WHO, 2017).

The complementary feeding period, which typically extends between 6 and 23 months of age, is a vulnerable period for growth faltering, micronutrient deficiencies, and common childhood illnesses (UNICEF, 2012). This is especially true in low‐income countries where access to fortified and hygienic complementary foods is limited. As infants gradually transit from exclusive breastfeeding to family food, they frequently get exposed to unhygienic, monotonous, and nutrient‐poor complementary foods (Gebremedhin, 2019). Analysis of multiple demographic and health surveys (DHS) indicated that the prevalence of anemia among children 6–23 months of age in SSA is as high as 75% and the consumption pattern of iron‐rich or iron‐fortified is very low (Gebremedhin, 2019; Prieto‐Patron, Van der Horst, Hutton, & Detzel, 2018).

During early childhood period, anemia and iron deficiency anemia cause multiple consequences including death due to severe anemia, impaired physical and psychomotor developments, weakened cell‐mediated immunity, and increased susceptibility to infectious diseases (Cherayil, 2010; Larson, Phiri, & Pasricha, 2017; UNICEF & WHO, 2011). A study based on data from multiple African countries estimated that each 1 g/dl increase in the population hemoglobin concentration is associated with a 24% decline in the risk of infant mortality (Scott, Chen‐Edinboro, Caulfield, & Murray‐Kolb, 2014). World Health Organization (WHO) recommends that children 6–23 months living in areas where the prevalence of anemia is above 40% should daily receive 10–12.5 mg elemental iron supplementation for 3 consecutive months per year (WHO, 2016).

Previous studies conducted in Malawi indicated that anemia was extremely common among preschool children (Calis et al., 2008; National Statistical Office, 2017). The recent national DHS found that in 2015, 62% of preschool children were anemic and the prevalence exceeded 80% among children younger than 18 months of age (National Statistical Office, 2017). Underlying risk factors of anemia among Malawian children include the following: iron, vitamin B‐12 and vitamin A deficiencies, infectious diseases including malaria, HIV infection and hookworm infestation, and genetic glucose‐6‐phosphate dehydrogenase deficiency (Calis et al., 2008). Rural place of residence, maternal illiteracy, and low household socioeconomic status were also associated with anemia (National Statistical Office, 2017).

Despite the exceptionally high burden of anemia among infants and young children (6–23 months of age) in SSA (Gebremedhin, 2019; Prieto‐Patron et al., 2018), the existing literature is primarily focused on the entire under five children and most studies only explored the sociodemographic determinants of anemia. Accordingly, the underlying causes of anemia and their epidemiological significance in children 6–23 months in SSA have not been adequately elucidated. This study, based on the secondary data of the Malawi Micronutrient Survey (MMS)—2015/2016, evaluated the contribution of multiple risk factors including micronutrient deficiencies, inflammation markers, genetic disorders, and infant feeding practices, to the hemoglobin level of children 6–23 months of age.

## 2 METHODS

### 2.1 Design and study subjects

**FIGURE 1:**
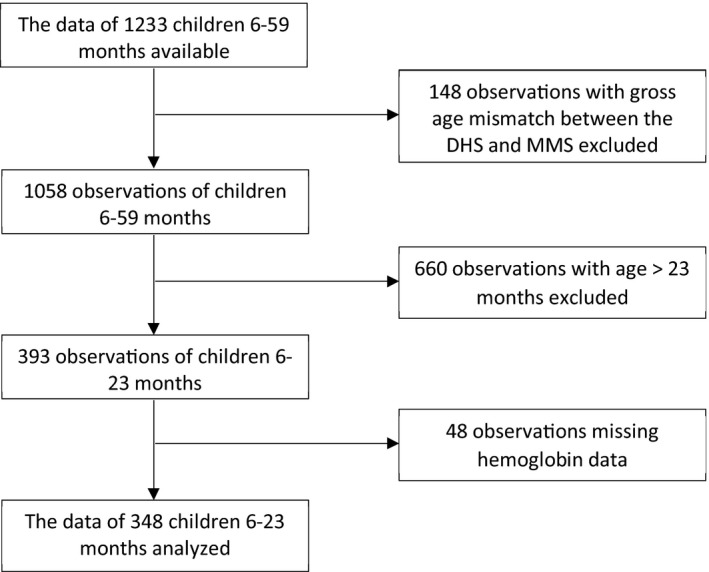
Flowchart of the study

This cross‐sectional study was conducted based on the secondary data of MMS, which was carried out between December 2015 and February 2016 on a subsample of households selected for the 2015–2016 Malawi DHS. The MMS collected diverse information relevant to anemia in multiple age groups including preschool children (6–59 months). For this specific study, the data of infant and young children 6–23 months were analyzed. The exclusion criteria were as follows: missing hemoglobin or child's age data, not listed in the original DHS dataset and, having age mismatch between the MMS and DHS datasets. Ultimately the data of 348 eligible infants and young children were analyzed (Figure 1).

### 2.2 Sample size

As the study was conducted based on secondary data, priory sample size estimation was not made. However, the adequacy of the available sample size for identifying predictors of hemoglobin was evaluated by G*power program (Faul, Erdfelder, Buchner, & Lang, 2009) using an approach suitable for multiple linear regression analysis. The specifications made during the computation were as follows: two‐tailed test, 80% power, 95% confidence level, medium effect size (Cohen's *f* ^2^ = 0.15), and 19 predictors in the regression model. In the post hoc sample size estimation, medium effect size was assumed because small effect size may not be clinically relevant and large effect size inflates type II error.

### 2.3 Sampling procedure and data collection approach of the primary survey

The sampling procedures of the Malawi DHS and MNS 2015–2016 are described in their respective published reports (National Statistical Office, 2017; National Statistical Office, CDC, & Emory University, 2017). In general, the DHS employed two‐stage cluster sampling approach designed to provide representative data at national, district, and place of residence (urban–rural) levels. Initially, 850 enumeration areas (EAs)—173 in urban and 677 in rural areas—were selected using probability proportional to size (PPS) sampling method. On average, each EA comprised 235 households. Among these EAs, 105 EAs—35 each from the northern, central, and southern regions of the country—were randomly selected for the micronutrient survey. Within each EA, an exhaustive listing of households was made, 20–22 households were randomly drawn from each EA, and all the available preschool children in the selected households were included in the micronutrient survey.

Data were collected from the mothers of the index children. The MMN survey collected diverse information relevant to anemia. Comprehensive sociodemographic data were collected using the standard DHS questionnaire. Occurrences of common childhood illnesses (cough, fever, diarrhea) in the preceding 2 weeks were evaluated based on the reports of the caregivers. Infant and young child feeding practices were assessed using an approach compatible with the existing WHO standard (WHO, 2008).

Furthermore, information pertaining to iron biomarkers (serum ferritin and soluble transferrin receptor [sTfR]), serum zinc, retinol binding protein (RBP), plasma selenium, inflammation markers (C‐reactive protein [CRP], and α‐1 acid glycoprotein [AGP]), genetic blood disorders pertinent to anemia (sickle cell, alpha‐thalassemia, glucose‐6‐phosphate dehydrogenase [G6PD] deficiency), malaria, and hematuria status, was collected. Hemoglobin was determined in the field using Hemocue 301^®^ and was adjusted for altitude (CDC, 1998). RBP, inflammation markers, ferritin, and sTfR were measured using ELISA. Inherited blood disorders were determined from dried blood spot using PCR and serum zinc was measured via atomic emission spectrometry. Malaria and hematuria diagnosed in the filed using rapid tests (National Statistical Office et al., 2017).

### 2.4 Variables of the study

The dependent variable of the analysis was hemoglobin level adjusted for altitude. The independent variables include the following: age, sex, and breastfeeding status of the child; child's dietary diversity as measured by the standard 7‐food groups scale (WHO, 2008); illness of the child (malaria, hematuria, fever, cough, and diarrhea); receiving deworming treatment in the past 6 months, altitude of the EA, iron status as measured by both ferritin and sTfR levels, serum zinc, RBP, plasma selenium, CRP, and AGP levels and the presence of the aforementioned three genetic disorders relevant to anemia. The independent variables were selected in consideration of relevant literature including the conceptual framework on the immediate causes of anemia proposed by Pasricha, Drakesmith, Black, Hipgrave, and Biggs (2013).

### 2.5 Data analysis

The data analysis was performed using STATA/SE 14.0 program. The DHS and MMS datasets for preschool children were separately downloaded from the DHS program website (The DHS Program, 2019) and merged using individual ID numbers. All continuous variables were assessed for normality using histogram and Kolmogorov–Smirnov test. Normally distributed covariates were described using mean and standard deviation (*SD*) whereas skewed variables summarized via median and interquartile range (IQR). Box‐and‐whiskers plot was used for identifying extreme outlier values and such values (scores beyond 3*IQR on both sides of the whiskers) were trimmed to the next acceptable value. Data were analyzed using weighted analysis approach based on the sampling weight fractions provided in the dataset. However, minor linearization correction was applied for keeping the weighted and unweighted sample sizes equal. Household wealth quintile was computed using principal component analysis (PCA) as commonly done in DHS. The hemoglobin level was compared across categories of the independent variables using independent *t* test or one‐way analysis of variance (ANOVA). The statistical assumptions of the tests were evaluated following standard approaches.

The association between hemoglobin and 19 predictors was assessed using multiple linear regression analysis with robust standard errors. Mixed‐effects model was not considered because the number of observations per cluster was too few for many of the clusters. All the 19 independent variables were entered into one multivariable model using the “enter” approach. The number of covariates in the model was not reduced because the sample size per covariate ratio was within the acceptable range (15–20 observations per variable; Austin & Steyerberg, 2015). The presence of multicollinearity among the covariates was assessed using variance inflation factor (VIF) and found to be within the acceptable range (VIF < 10). The VIF ranged from 1.10 for wasting to 1.82 to CRP. Partial plots were used for evaluating the normality and homoscedasticity of error terms. The goodness of fit was measured by adjusted‐*R* ^2^ value and *F*‐statistic. The relative contribution of each of the predictor to the model was evaluated using delta‐*R* ^2^ value, as determined by nested regression approach. Two independent variables (hematuria and sickle cell disease) were dropped from the regression analysis because they had small number of observations for some of their categories (only one child was affected by sickle cell disease and eight had positive dipstick test for hematuria).

The decision to enter the continuous versus categorical, or the natural versus transformed form of a specific covariate was made based on the delta‐*R* ^2^ value of each version of the variables in consideration of the assumptions of the model. For example, in the final model the continuous rather than categorical forms of CRP and AGP were entered because the earlier provided a better fit. Likewise, the natural, rather than the log‐transformed form of ferritin was used because it yielded better fit without violating the assumptions of the model. When categorical variables were fitted, clinically meaningful cutoffs were adopted.

## 3 RESULTS

### 3.1 Sociodemographic characteristics of the respondents

Table 1 presents the sociodemographic characteristics of the mothers and the study children. Ninety percent of the respondents were selected from rural areas, and 93.4% had formal education. The mean (±*SD*) age was 26.5 (±6.2) years, and 86.4% were below 35 years of age. Regarding the infants and young children, their average (±*SD*) age was 14.6 (±5.0) months and boys (54.7%) were slightly over represented than girls (Table 1).

**TABLE 1:** Sociodemographic characteristics of the respondents, Malawi, 2015–2016

Variable (*n* = 348)	Frequency	Percentage
Maternal education		
No formal education	22	6.4
Primary education	247	71.1
Secondary education	75	21.4
Higher education	4	1.0
Place of residence		
Urban	35	10.1
Rural	313	89.9
Maternal age (years)		
15–24	154	44.4
25–34	146	42.0
35 or above	47	13.6
Marital status		
Married/living together	295	84.7
Separated/divorced	33	9.6
Widowed	6	1.7
Not ever married	14	4.0
Household wealth index		
Poorest	85	24.5
Poorer	77	22.1
Middle	75	21.7
Richer	68	19.6
Richest	43	12.2
Child's age (months)		
6–11	104	29.9
12–17	134	38.5
18–23	110	31.6
Child's sex		
Boy	190	54.7
Girl	158	45.3
Birth order		
1st	103	29.7
2nd to 4th	166	47.7
5th or above	78	22.5

### 3.2 Inflammation, illness, and genetic abnormalities

Inflammation appeared to be very common among infant and young children in Malawi. The median (IQR) of CRP concentrations was 1.8 (0.6–6.0) mg/L, and 28.3% had elevated levels (>5 mg/L). Similarly, the median AGP was 1.0 (0.0–2.0) g/L and 53.9% had elevated AGP (>1 g/L). In combination, rise in both of the inflammation markers was observed in 25.6% of the children, whereas 30.7% had inflammation based on either of the markers.

During the survey, 23.6% of the children tested positive for malaria whereas 3.3% had a positive dipstick test for hematuria. Nearly one‐third reportedly had fever (30.4%), diarrhea (33.9%) and cough (34.1%) in the preceding 2 weeks.

The MMS also assessed the prevalence of three genetic conditions—alpha‐thalassemia, sickle cell disease, and G6PD deficiency—that have relevance to anemia. In terms of children affected, G6PD deficiency was relatively common (11.8% affected, 12.4% carrier) followed by alpha‐thalassemia (7.4% affected, 34.8% carrier) and sickle cell disease (0.2% affected, 7.7% carrier).

### 3.3 Breastfeeding and complementary feeding status

At the time of the survey, 82.9% of the children were receiving breastmilk. However, small proportions (18.8%) met the minimum recommend dietary diversity—receiving from 4 or more food groups from the standard seven food groups—in the preceding day of the survey. The majority of the children consumed grains, roots, or tubers (70.5%) and vitamin A rich fruits and vegetables (71.1%) in the reference day. Conversely, smaller proportions consumed other fruits and vegetables (23.1%) and legumes and nuts (16.0%). Consumption pattern of animal sources foods including flesh foods (33.8%), eggs (7.4%), and dairy products excluding breastmilk (4.4%) was relatively lower.

### 3.4 Anthropometric status, micronutrient deficiencies, and anemia

Table 2 presents the anthropometric and micronutrient status of children 6–23 months in Malawi. About a quarter (23.4%) of children were stunted and 5.5% were wasting. More than three‐fourth (77.0%) had selenium deficiency (plasma selenium <70 ng/ml) (Combs, 2015) and 57.1% were zinc‐deficient based on cutoff that has taken into consideration time of blood collection and fasting status of the subjects (King et al., 2016). On the basis of inflammation‐adjusted ferritin levels (Suchdev et al., 2016), the prevalence of iron deficiency (42.0%) was also very high. Conversely, vitamin A deficiency was rare (3.2%) (Table 2).

**TABLE 2:** Anthropometry and micronutrient status of infants and young children 6–23 months in Malawi, 2015–2016

Index (*n* = 348)	Mean (*SD*)	Proportions below standard cutoffs (%)^a^
Height‐for‐age (*z*‐score)	−1.20 (1.37)	23.4
Weight‐for‐age (*z*‐score)	−0.53 (1.26)	10.7
Weight‐for‐height (*z*‐score)	−0.08 (1.21)	5.5
Retinol binding protein (µmol/L)	0.88 (0.23)	3.2
Serum zinc (µg/dl)	59.93 (16.93)	57.1
Plasma selenium (ng/ml)	56.85 (22.85)	77.0
Serum ferritin (µg/L) (unadjusted)	23.04 (11.38–41.04)^b^	26.0
Serum ferritin (µg/L) (adjusted)^c^	13.94 (8.09–24.30)^b^	42.0
Hemoglobin (g/dl)	10.98 (1.27)	43.9
Iron deficiency anemia^d^	–	15.2

^a^ < −2 *z*‐score for the three anthropometric indices; <0.46 μmol/L for retinol binding protein (calibrated to equal retinol <0.7 μmol/L), <65 or <57 μg/dl for zinc deficiency depending of time of data collection; <70 ng/ml for plasma selenium; <12 µg/L for serum ferritin; <11 g/dl for hemoglobin.

^b^ Median and interquartile range.

^c^ Adjusted for inflammation using the internal regression correction approach.

^d^ Inflammation corrected iron deficiency plus anemia.

The mean (±*SD*) hemoglobin concentration among the children was 10.9 (±1.3) g/dl and nearly half (43.9%) had low hemoglobin level (<11 g/dl) indicating anemia. In terms of the degree of severity, 24.5% had mild (10–10.9 g/dl), 18.7% moderate (7–9.9 g/dl), and 0.8% severe (<7 g/dl) anemia. In consideration of inflammation‐adjusted serum ferritin levels (Suchdev et al., 2016), 15.2% had concomitant iron deficiency and anemia, suggesting iron deficiency anemia. The mean (±*SD*) sTfR concentration was 14.6 (±9.3) mg/L and 76.9% of the children had high sTfR (>8.3 mg/L) implying iron‐deficient erythropoiesis (Rohner et al., 2017).

Table 3 compares the hemoglobin concentration across selected independent factors using independent *t* test or ANOVA. Very strong statistically significant associations (*p* < .001) were observed between hemoglobin and, iron, vitamin A, iron‐deficient erythropoiesis and inflammation status. Statistically significant associations were also observed with child's age, alpha‐thalassemia, and wasting status (Table 3).

**TABLE 3:** Comparison of hemoglobin concentration across pertinent independent variables, Malawi, 2015–2016

Variable (*n* = 348)	Mean (±*SD*) hemoglobin (g/dl)	*p*‐value
Age (months)		
6–11	10.7 (±1.1)	.049*
12–17	11.0 (±1.3)	
18–23	11.2 (±1.3)	
Sex (*n* = 348)		
Boy	10.9 (±1.3)	.057
Girl	11.1 (±1.2)	
Wasting		
Normal	11.0 (±1.3)	.042*
Wasted	10.4 (±1.6)	
Stunting		
Normal	11.0 (±1.2)	.810
Stunted	11.0 (±1.3)	
Zinc status		
Normal	11.1 (±1.3)	.184
Deficient	10.9 (±1.2)	
Selenium status		
Normal	11.0 (±1.1)	.605
Deficient	11.0 (±1.2)	
Iron status		
Normal	11.2 (±1.2)	<.001*
Deficient	9.9 (±0.8)	
Iron‐deficient erythropoiesis		
No	11.6 (±1.1)	<.001*
Yes	10.8 (±1.2)	
Vitamin A status		
Normal	11.0 (±1.2)	<.001*
Deficient	9.3 (±1.4)	
CRP level		
Normal	11.2 (±1.1)	<.001*
Elevated	10.4 (±1.4)	
AGP level		
Normal	11.3 (±1.1)	<.001*
Elevated	10.7 (±1.3)	
Currently breastfeeding		
Yes	11.0 (±1.2)	.063
No	10.7 (±1.4)	
Dietary diversity		
Suboptimal	10.9 (±1.2)	.327
Optimal	11.1 (±1.3)	
Malaria		
Positive	10.4 (±1.3)	<.001*
Negative	11.1 (±1.4)	
Hematuria		
Positive	11.0 (±1.3)	.881
Negative	10.9 (±1.2)	
Iron‐deficient erythropoiesis		
No	11.6 (±1.1)	<.001*
Yes	10.8 (±1.2)	
Alpha‐thalassemia		
Unaffected	10.9 (±1.3)	.003*
Carrier	11.2 (±1.3)	
Affected	10.2 (±0.9)	
G‐6‐PD deficiency		
Unaffected	10.9 (±1.2)	.207
Carrier	11.3 (±1.3)	
Affected	10.8 (±1.4)	

* Statistically significant association at *p*‐value of .05.

### 3.5 Predictors of hemoglobin status among infants and young children 6–23 months

Table 4 summarizes the outputs of simple and multiple linear regression analyses on the relationship between 19 predictors and hemoglobin status of infants and young children. In the multiple model, ten variables, including the two iron biomarkers (Ferritin and sTfR), plasma selenium level, inflammation markers (CRP, AGP), malaria status, history of fiver in the preceding 2 weeks, alpha‐thalassemia, age of the child and altitude of the cluster, emerged as significant predictors of hemoglobin (*p* < .05).

**TABLE 4:** Predictors of hemoglobin status among infants and young children 6–23 months in Malawi, 2015–2016

Independent variables (*n* = 348)	Coding scheme/range (unit)	Bivariable models			Multivariable model^a^			
		Regression coefficient (95% confidence interval)	*t*	*p*‐value	Regression coefficient (95% confidence interval)	*t*	*p*‐value	Delta‐*R* ^2^ (%)
Age (months)	6–23	0.037 (0.008, 0.067)	2.51	.012*	0.038 (0.013, 0.063)	3.01	.003*	2.1
Altitude (m)	52–1626	0.0003 (−0.0001, 0.0008)	1.50	.136	0.0006 (0.0001, 0.0010)	2.44	.015*	1.5
Ferritin (µg/L)	2.63–266.07	−0.004 (−0.009, 0.000)	−1.88	.061	0.005 (0.000, 0.009)	2.06	.041*	1.7
sTfR (mg/L)	5.52–60.0	−0.045 (−0.078, −0.011)	−2.63	.009*	−0.050 (−0.075, −0.025)	−3.90	<.001*	9.1
RBP (mg/L)	0.26–1.66	1.272 (0.569, 1.975)	3.56	<.001*	0.637 (−0.036, 1.310)	1.86	.063	1.3
Plasma selenium (ng/ml)	12.53–165.40	0.007 (0.001, 0.014)	2.27	.024*	0.007 (0.001, 0.013)	2.34	.020*	1.3
Serum zinc (µg/dl)	18.75–168.75	0.005 (−0.004, 0.015)	1.06	.288	0.002 (−0.007, 0.011)	0.43	.669	0.1
Malaria	0 = no; 1 = yes	−0.695 (−0.295, −1.095)	−3.42	.001*	−0.430 (−0.046, −0.814)	−2.20	.029*	1.5
Fever	0 = no; 1 = yes	−0.324 (−0.674, 0.026)	−1.82	.069	−0.392 (−0.684, −0.101)	−2.66	.008*	1.9
Diarrhea	0 = no; 1 = yes	0.185 (−0.157, 0 0.527)	1.07	.287	−0.130 (−0.452, 0.192)	−0.80	.425	0.2
Deworming	0 = no; 1 = yes	0.316 (0.771, −0.137)	1.37	.172	0.354 (−0.032, 0.739)	−1.81	.072	0.7
AGP (g/L)	0.29–4.00	−0.477 (−0.669, −0.285)	−4.89	<.001*	−0.339 (−0.584, −0.094)	−2.72	.007*	3.3
CRP (mg/L)	0.0–121.13	−0.020 (−1.190, −0.422)	−2.66	.008*	−0.014 (−0.024, −0.004)	−2.88	.004*	1.9
Alpha‐thalassemia	0 = Unaffected^×^; 1 = affected	−0.797 (−1.257, −0.337)	−3.41	.001*	−0.753 (−1.133, −0.372)	−3.90	<.001*	2.5
G‐6‐PD deficiency	0 = Unaffected^×^; 1 = affected	−0.203 (−0.764, 0.357)	−0.71	.476	−0.065 (−0.413, 0.283)	−0.37	.713	0.0
Currently breastfeeding	0 = no; 1 = yes	0.338 (−0.060, 0.737)	1.67	.096	0.125 (−0.222, 0.472)	0.71	.478	0.1
Dietary diversity	0 = suboptimal; 1 = optimal	0.172 (−0.252, 0.597)	0.80	.426	−0.177 (−0.539, 0.184)	−0.97	.334	0.2
Wasting	0 = not wasted; 1 = wasted	−0.631 (−1.563, 0.302)	−1.33	.184	−0.213 (−0.785, 0.359)	−0.73	.464	0.1
Stunting	0 = not stunted; 1 = stunted	0.040 (−0.305, 0.385)	0.23	.819	0.150 (−0.144, 0.444)	1.01	.315	0.3

Abbreviations: AGP, α‐acid glycoprotein; CRP, C‐reactive protein; G‐6‐PD, glucose‐6‐phosphate dehydrogenase; RBP, retinol binding protein; sTfR, soluble transferrin receptor.

^a^ Multivariable linear regression model containing all the 19 covariates; *r* ^2^ = 47.6%; adjusted *r* ^2^ = 43.1%.

* Statistically significant association at *p*‐value of .05.

^×^ Unaffected including carries.

In the final model, unit changes in serum ferritin and sTfR levels were associated with significant 0.01 g/dl increase (*p* = .041) and 0.05 g/dl decline (*p* < .001) in the hemoglobin concentrations, respectively. Similarly, each 1‐ng/ml increase in plasma selenium was met with 0.007 g/dl (*p* = .02) increase in hemoglobin. Children with positive test for malaria on average had 0.43 g/dl lower hemoglobin concentration than their counterparts (*p* = .029). Similarly, the hemoglobin level among children who had fever in the last 2 weeks was significantly lower by 0.39 g/dl than afebrile children (*p* = .008). Children affected by alpha‐thalassemia on average had 0.75 g/dl lower hemoglobin concentration than unaffected or carrier children (*p* < .001). Hemoglobin showed significant negative relationships with AGP (*β* = −.339, *p* = .007) and CRP (*β* = −.014, *p* = .004) levels and significantly positive association with child's age in months (*β* = .038, *p* = .003) and altitude in meters (*β* = .001, *p* = .015).

Among the remaining variables, RBP concentration and history of deworming in the last 6 months showed marginally insignificant associations with hemoglobin (.1 > *p* > .05); whereas serum zinc level, child anthropometric indicators, breastfeeding status, dietary diversity, recent history of diarrhea, and G6PD deficiency were not associated with hemoglobin.

As described earlier, the relative contribution of each predictor to the variability explained by the model was assessed by delta‐*R* ^2^ value. sTfR had the highest contribution (9.1%) to hemoglobin variability; followed by AGP (3.3%), alpha‐thalassemia (2.5%), child's age (2.1%), CRP (1.9%), and fever (1.9%) (Table 4).

## 4 DISCUSSION

In SSA, infants and young children 6–23 months of age take the highest burden of anemia. However, the underlying causes had not been adequately explored. This analysis identified multiple risk factors of anemia, with their relative epidemiologic significance, among Malawian children 6–23 months.

In this study, two iron biomarkers (sTfR and ferritin) found to be significant predictors of hemoglobin. While ferritin displayed marginally significant relationship, sTfR emerged as the strongest predictor with the highest delta‐*R* ^2^ contribution to the model. The weak association observed between ferritin and hemoglobin can be due to the high prevalence of inflammation and the acute phase reactant nature of ferritin. Though ferritin is a sensitive and valuable index of iron nutrition, its utility in settings with high prevalence of inflammation is limited (Dignass, Farrag, & Stein, 2018; Nemeth & Ganz, 2014).

Convincing evidence exists that sTfR is more resistant to inflammation and it is an important biomarker of iron‐deficient erythropoiesis in hospitalized patients and populations with high prevalence of inflammation, including preschool children (Beguin, 2003; Ragab, Ibrahim, Eid, Kotb, & Konsowa, 2002; Rohner et al., 2017). The strong association between hemoglobin and sTfR levels, the high prevalence of iron‐deficient erythropoiesis (77%), and the low consumption of flesh foods (34%) suggested that iron deficiency is the foremost important cause of anemia in young children in Malawi. The regression analysis might even have underestimated the significance of iron due to the statistical adjustment of sTfR for ferritin. Nevertheless, both were kept in the equation with the intension of maximizing the goodness of fit of the model.

A negative association was also observed between hemoglobin and inflammation, and in combination, the two inflammation markers had the second highest delta‐*R* ^2^ contribution (about 5%) to the model. This is consistent to understand that anemia of inflammation (AI) is the second most common cause of anemia worldwide, next to iron deficiency anemia (Ganz & Nemeth, 2009; Nemeth & Ganz, 2014). It has been hypothesized that, during inflammation, the pro‐inflammatory cytokine IL‐6 increases hepcidin to cause iron sequestration that in turn leads to iron‐restricted erythropoiesis. Further, inflammation may induce destruction of erythrocytes and suppress maturation of precursors of erythropoiesis (Fraenkel, 2017; Ganz & Nemeth, 2009; Nemeth & Ganz, 2014).

Selenium level was found to be an independent predictor of hemoglobin. The association between selenium and hemoglobin has not been extensively investigated in humans before. Yet, the available cross‐sectional studies among school‐aged children (Houghton, Parnell, Thomson, Green, & Gibson, 2016; Nhien et al., 2008), adults (Larvie, Doherty, Donati, & Armah, 2019), and the old (Bates, Thane, Prentice, & Delves, 2002; Semba et al., 2009) reported significant associations. Though the possible mechanism by which selenium deficiency causes anemia has not been fully elucidated, possible pathways including selenium deficiency‐inducted oxidative stress, chronic inflammation, and increased expression of heme oxygenase enzyme had been suggested (Petkova‐Marinova, Ruseva, & Atanasova, 2017; Semba et al., 2009). Low serum zinc and hepcidin may also mediate the effect of selenium on hemoglobin (Houghton et al., 2016; Larvie et al., 2019).

The analysis showed that hemoglobin level tends to increase with rising altitude. Conclusive evidence exists that hemoglobin rises at high altitude as a physiological response to elevation‐induced hypoxia (CDC, 1998; WHO, 2011). Yet more surprising is the association was sustained despite the use of altitude adjusted hemoglobin values recommended by CDC (1998). The unexpected finding might be due to a couple of reasons. First, systematic difference many exists in the basic characteristics of populations residing at different altitudes; hence, the observed association can be the indirect reflection of variation in other unaccounted parameters relevant to hemoglobin. For instance, the burden of infectious and parasitic diseases might be elevated in the lowlands to cause higher prevalence of anemia. Second, as the altitude correction formula recommended by CDC was developed exclusively based on the data of the US population, the correction might not be entirely applicable to other settings. Previous studies have recommended for revisions of the WHO or CDC altitude corrections due to concerns of over‐ or underadjustment (Ocas‐Cordova, Tapia, & Gonzales, 2018; Sharma, Addo, Mei, & Suchdev, 2019).

The study found that malaria was very common among infants and young children (24%) in Malawi and children tested positive for malaria on average had 0.4–0.7 g/dl lower hemoglobin concentrations. This is consistent to the understanding that malaria causes anemia through multiple pathways including hemolysis of erythrocytes, suppressing of erythropoiesis, and maldistribution of iron (Helleberg et al., 2005; White, 2018). Further, malaria infection increases hepcidin that reduces absorption of iron in the blood (Prentice, Ghattas, Doherty, & Cox, 2007). In this analysis, we might have even underestimated the contribution of malaria to hemoglobin due to the statistical adjustment of malaria to multiple inflammation markers, fever, and other symptoms of the disease.

In this study, child's age showed an independent negative association with hemoglobin status. This is consistent to the findings of studies conducted in other low‐income countries (Huang et al., 2018; Mohammed, Habtewold, & Esmaillzadeh, 2019). Prolonged exclusive breastfeeding, delayed initiation of complementary foods, and initiating complementary feeding with poorly diversified diet could contribute for the lower hemoglobin levels in infants. Further, a study that analyzed hepcidin pattern among young Kenyan children concluded that hepcidin—a negative regulator of iron absorption—declines throughout infancy and reaches the lowest level between 1 and 2 years of age, suggesting iron absorption is higher in the second year of age than during infancy (Atkinson et al., 2015).

The main strength of this study is the fact that the original survey gathered information on many relevant variables including micronutrient indicators, inflammation markers, and inherited blood disorders that are not very frequently measured in large‐scale community‐based surveys. On the other hand, a number of limitations need to be recognized. First, due to the cross‐sectional and observational nature of the study, the causal inference is likely to be weak the possibility of egg–chicken dilemma cannot be excluded. Second, with the intension of maximizing the goodness of fit of the model, many independent variables, including multiple markers of iron and inflammation, were fitted. This may have caused over‐adjustment bias and may underestimate the strength of association with hemoglobin. Further, adjustment of child feeding indicators (breastfeeding status and dietary diversity) for micronutrient status can underestimate their significance for anemia because improving micronutrient status is among the major pathways through which optimal child feeding prevents anemia. Finally, despite the fact that many covariates had been measured and adjusted in the study, some relevant predictors (e.g., B‐12 and folate status, dysentery) were still missing. This might have lowered the goodness of fit of the model and induced residual confounding.

## 5 CONCLUSION

Iron deficiency and inflammation showed major contributions to the hemoglobin status of infants and young children 6–23 months in Malawi, possibly suggesting iron deficiency and anemia of infection are the major underlying causes of anemia in the country. Other factors that emerged significant predictors of hemoglobin include alpha‐thalassemia, malaria, other febrile illnesses, and serum selenium level. The assortation between plasma selenium and hemoglobin status should be investigated through randomized controlled trials.

## CONFLICT OF INTEREST

The author declares that he does not have any conflict of interest.

## ETHICAL APPROVAL

This original study was approved by the Institutional Review Board of Malawi National Health Sciences Research Committee.

## INFORMED CONSENT

In the original study, written informed consent was obtained from all study participants.
